# Challenge Studies to Determine the Ability of Foods to Support the Growth of *Listeria monocytogenes*

**DOI:** 10.3390/pathogens7040080

**Published:** 2018-10-05

**Authors:** Karen Hunt, Marjorie Blanc, Avelino Álvarez-Ordóñez, Kieran Jordan

**Affiliations:** Teagasc Food Research Centre, Moorepark, Fermoy, Co. Cork, Ireland; Karen.hunt@teagasc.ie (K.H.); marjorie.blanc@teagasc.ie (M.B.); aalvo@unileon.es (A.Á.-O.)

**Keywords:** *Listeria monocytogenes*, growth, challenge study, food

## Abstract

*Listeria monocytogenes* is a foodborne pathogen that causes listeriosis, a relatively rare, but potentially fatal, disease, with a mortality rate of 20–30%. In general, European Regulations require the absence of *L. monocytogenes* in five samples of 25 g before the food has left the producer, but if the food has been demonstrated not to support the growth of *L. monocytogenes*, up to 100 cfu g^−1^ are allowed in the food (except for foods for infants or medical purposes) during its shelf-life under reasonably foreseeable storage conditions. It is important for food producers to determine if their food supports the growth of *L. monocytogenes*. The European Union Reference Laboratory for *L. monocytogenes* published a Technical Guidance document for conducting shelf-life studies on *L. monocytogenes* in ready-to-eat foods in June 2014. Primarily based on the EURL guidance document for conducting challenge studies, the ability of cheese (feta and soft goat’s milk cheese), cold-smoked salmon, coleslaw, and pork pate to support the growth of *L. monocytogenes* was determined using a starting inoculum of approximately 100 cfu g^−1^. The cheese and pork pate were incubated at 8 °C for 14 days; the smoked salmon was incubated at 6 °C for 5 days and 8 °C for 9 days; and the coleslaw was incubated at 8 °C for 7 days and 12 °C for 14 days. The results showed that the smoked salmon and pork pate supported growth, while coleslaw and cheese did not. From this study, it is evident that there are factors in food other than pH, water activity, and total bacterial count (TBC) that can inhibit the ability of *L. monocytogenes* to grow in food.

## 1. Introduction

*Listeria monocytogenes* is widely distributed in the environment, being found in soil, water, and plant material, among other things [1]. It can, therefore, contaminate the food processing environment unless stringent efforts are in place to prevent such contamination. *L. monocytogenes* can survive for long periods of time in a seemingly hostile environment, such as a food processing facility. This is partially due to its ability to survive various stresses, such as sanitisers, pH, and temperature [2,3], and its ability to form a biofilm [4,5], leading to persistence [6]. Thus, it is a concern for the food industry. If present in the food processing environment, cross-contamination is a possible route of food contamination [7], where it can be an issue, particularly for ready-to-eat (RTE) foods.

With the absence of cooking, or a similar bacterial inactivation step, in the production of RTE foods, *L. monocytogenes* can persist, and if conditions become favourable, it may grow to numbers high enough to cause infection. According to the European food law (Regulation 2073/2005), in the case of foodstuffs that can support the growth of *L. monocytogenes*, food business operators (FBOs) must demonstrate its absence in five samples of 25 g, or, in foods for special dietary purposes, its absence in 10 samples of 25 g. In contrast, in those foodstuffs that do not support the growth of *L. monocytogenes*, a maximum level of 2 Log cfu/g is allowed during the shelf-life of the food. Consequently, food processors must demonstrate if their products support the growth of *L. monocytogenes.* If the inability of *L. monocytogenes* to grow in the food has not been demonstrated in a food challenge study, then growth is presumed. Therefore, it is important for RTE food producers to determine the ability of each specific food to support the growth of *L. monocytogenes.*

To predict the growth of *L. monocytogenes* in foods, the use of predictive microbiology software, such as Combase [8,9] or Pathogen Modelling Programme [10], among others, have been described. These software programmes consider several factors, such as pH, temperature, and water activity (a_w_), to predict the growth of *L. monocytogenes*. However, not all factors can be considered and so predictions may be inaccurate, as shown by Schvartzman et al. [11].

To support food producers in doing challenge studies, the European Union Reference Laboratory (EURL) for *L. monocytogenes* published a Technical Guidance document for conducting shelf-life studies on *L. monocytogenes* in ready-to-eat foods in 2008. This guidance was revised and the revision published in 2014 [12]. Factors, such as temperature, inoculum level, preparation, competing microflora, and water activity, are considered in making recommendations for undertaking challenge studies. The document emphasises the fact that a challenge study needs to be undertaken for each specific food as the results from one food cannot be extrapolated to other foods.

There are several recent studies on challenge studies to determine the ability of foods to support the growth of *L. monocytogenes* [13,14,15]. While some of these studies have broadly followed the EURL guidelines [13], others have not [14,15]. The comparison of the results from one study to the next is not possible unless the same methods are used for the challenge study. Other studies have attempted to model the growth of *L. monocytogenes* in food [16,17]. Such modelling studies require single strains, a constant storage temperature, and more data points (among other things). Therefore, the EURL guidelines used in the current experiments for challenge studies are not appropriate for modelling.

The objective of this study was to assess the growth potential (δ) of *L. monocytogenes* on cold-smoked salmon, cheese, pork pate, and coleslaw, using the guidelines published by the EURL. 

## 2. Results

Prior to conducting the challenge studies, all foods were negative for *L. monocytogenes* (by enrichment), except for one batch of smoked salmon, which was not used for the challenge studies. The target inoculum of approximately 100 cfu g^−1^ was achieved for all foods.

According to the EURL guidelines, a food has the potential to support growth if one of the replicates has an increase in *L. monocytogenes* numbers of > log_10_ 0.5 cfu g^−1^. The results show that the cheeses and coleslaw did not support the growth of *L. monocytogenes* as in all batches tested, the numbers of *L. monocytogenes* in all the replicates of all the batches tested decreased in the first week and continued to decrease during the second week (Table 1).

On the other hand, pork pate and smoked salmon supported the growth of *L. monocytogenes* as at least one of the replicates showed > 0.5 log cfu g^−1^ difference between the numbers at the beginning of the experiment and the numbers at the end of the experiment (Table 1). For pork pate, growth was observed during the first week of storage so the experiment was stopped at that stage. The amount of growth in the pork pate for the first week was higher than that in the smoked salmon. The amount of growth on the smoked salmon from each manufacturer was similar (Table 1). 

As can be seen from Table 2, the growth of *L. monocytogenes* was supported in foods where the initial pH was greater than 6.0. In these foods, the pH decreased (although not significantly, *p* > 0.05) from about 6.2 to about 5.7 during storage, except for smoked salmon from manufacturer 1 where the pH did not drop below 6.0. In the cheese, where growth was not supported, the pH values were approximately 4.6 initially and decreased to 4.2–4.4. The pH of the coleslaw increased during the shelf-life of the product (not significantly, *p* > 0.05), although no growth occurred.

At the beginning of the experiments, all the values for water activity were above 0.96 and were similar. The water activity values decreased (not significantly, *p* > 0.05) during storage, but remained above 0.96, except for the feta cheese, which showed a significant decrease (*p* < 0.05) to 0.945 after storage, and smoked salmon from manufacturer 2, which showed a significant decrease (*p* < 0.05) to 0.950 after storage (Table 2). 

The initial total bacterial counts for the coleslaw and cheeses were about 7 log cfu g^−1^, whereas the initial counts were lower in the salmon and pork pate. During storage, there was no significant increase (*p* > 0.05) in the counts for coleslaw or the cheeses, whereas there was a significant increase (*p* < 0.05) in total counts for pork pate and smoked salmon during storage (Table 1).

## 3. Discussion

The results of this study show that the potential of food to support the growth of *L. monocytogenes* is dependent on the food type. Predictive microbiology can be used to give an indication of whether growth will be supported or not, but experiments must be undertaken in each food to definitively determine the ability of *L. monocytogenes* to grow in the food. For that reason, the EURL and other jurisdictions have published guidelines for undertaking challenge studies to determine the ability of food to support the growth of *L. monocytogenes* [12]. One of the important factors in these guidelines is the temperature at which the growth experiments are undertaken. The EURL guidelines recommend a temperature profile representing three phases, manufacturing, distribution and storage, and the consumer. For products with a shelf life of less than 21 days, the suggested temperatures are one-third of the shelf life at 8 °C, one-third at 12 °C, and one-third at 12 °C to represent each of the three phases, respectively. If there is national data to represent each of the phases, as in Ireland [18], or valid company specific data, this can also be used. Health Canada recommends that temperatures and times appropriate for the food commodity and its storage conditions should be used when designing challenge studies for specific commodities [19].

In this study, different time-temperature profiles were used for the foods tested. For smoked salmon, a temperature profile of 6 °C for one-third of the shelf-life and 8 °C for two-thirds of the shelf-life were used (following the manufacturer’s recommendation). For coleslaw, the default EURL guideline temperature profile was used (8 °C for 7 days and 12 °C for 14 days) and for cheese and pork pate, a constant temperature of 8 °C was used (as is used in challenge tests in the United Kingdom). Cold-smoked salmon is normally manufactured and distributed at 4 °C so the temperature profile used was considered by the manufacturer as reasonably foreseeable abuse conditions. Similarly, the temperature profile used for the cheese and pork pate was seen as reasonably foreseeable abuse conditions.

Water activity and pH and are important determinants of the ability of *L. monocytogenes* to grow in a food. The EU guidelines indicate that foods with a pH ≤ 4.4, a water activity of ≤ 0.920, or a combination of pH ≤ 5.0 and a water activity ≤ 0.940 will not support the growth of *L. monocytogenes* [12]. This was validated in the current study as the pH of the goat’s milk cheese was < 4.4 and no growth was observed. The pH of the feta cheese was about 4.6, and the water activity was 0.973 and, therefore, was theoretically capable of supporting growth. As no growth was observed, there were other factors in the cheese that inhibited growth. This result does show that predictive modelling has limitations in assessing the ability of foods to support the growth of *L. monocytogenes* [11].

Like other studies [20,21], cold-smoked salmon from both manufacturers supported the growth of *L. monocytogenes.* The source of *L. monocytogenes* on salmon can be from the raw material [22,23]. In such cases, cold-smoking the salmon will not necessarily inactivate *L. monocytogenes.* Cold-smoking processes differ in their manufacturing protocol and therefore each process needs to be tested for its impact on *L. monocytogenes*. Uyttendaele et al. [24] showed that at 4 °C, 13 of 25 samples of smoked fish did not support the growth of *L. monocytogenes*. Therefore, the results from the current study cannot be extrapolated to other smoked salmon (or other foods) from different manufacturers. Hot smoking of salmon will reduce *L. monocytogenes* to acceptable levels, and in that case, cross-contamination from the processing environment is the main concern [6,23].

The water activity of the cheese was above the theoretical limit for supporting growth, indicating that other factors in the cheese influenced the ability of the food to support growth. As it would be in cheese, the total bacterial count was relatively high at the start of the experiment, and this may have inhibited growth by producing antimicrobial compounds, for example. A recent study on the growth of *L. monocytogenes* in Queso Fresco cheese [25] showed that the cheese supported growth. In that study, the incubation temperature used was 4 °C and the inoculum level was approximately 3.7 log cfu g^−1^. The pH of the cheeses was above 6.0, and while the water activity was not given, the moisture content was about 50%. Therefore, the cheese would theoretically support growth, and was shown to support growth. However, as a different incubation temperature and inoculation level were used, or because of the different background microflora, the results cannot be compared with the current study.

The pH and water activity of the coleslaw indicated that the growth of *L. monocytogenes* was possible, so it is likely that the carrot in the coleslaw used in the current experiments contributed to the inhibition of the growth. Beauchat and Bracket [26] showed that raw carrot (whole of shredded) inhibited *L. monocytogenes*. In a previous study of the growth of *L. monocytogenes* in coleslaw, George and Levett [26] showed that, like the results of this study, at pH values below 6.0, no growth occurred at storage temperatures up to 15 °C. The inoculation concentration used can also influence the growth of *L. monocytogenes* in food [27,28], but in the case of inactivation, the results from different studies are somewhat comparable, and indicate that coleslaw does not support growth.

In the current study, the pH and water activity indicated that growth on pork pate was possible and growth was observed. The constituents of pate can vary greatly and therefore the ability of *L. monocytogenes* to grow in pate could also vary. In a study by Farber et al. [29], 16 different liver pâté formulations were made experimentally and all of them supported the growth of *L. monocytogenes*. In modelling the data, storage temperature was the only factor that influenced the growth rate. 

## 4. Materials and Methods

### 4.1. Strains Used

The strains used in the study were strain 6179, a persistent isolate from a cheese processing environment (isolated at the same facility for more than 10 years), strain 1020, which was isolated from raw bovine milk cheese, and strain 1382, which was from the EURL Lm strain collection. The strains were previously confirmed as *L. monocytogenes* by polymerase chain reaction (PCR) [30]; and independently grown in brain heart infusion (BHI) broth, which was incubated overnight at 37 °C. From this, 100 µL was transferred to 10 mL of BHI broth and incubated at 8 °C for six days, until the stationary phase was reached.

### 4.2. Inoculum Preparation

Each strain that was grown at 8 °C was independently serially diluted in maximum recovery diluent (MRD) from the initial cell count of 10^8^ cfu/mL and 10 mL of the final dilution of each culture was mixed to give a final inoculum of 30 mL at approximately 10^4^ cfu/mL, containing the three-strain mix. Because the strains grew at 8 °C, they were not incubated alongside the product, representing a minor deviation from the EURL guidelines. To ensure that the desired numbers were achieved, the cell numbers were confirmed by plate count using the ISO 11290-2 standard method [31].

### 4.3. Foods Used

Greek feta cheese (made from sheep’s milk) and pork liver paté were purchased in a local shop. Raw soft goat’s milk cheese, coleslaw, and smoked salmon from two manufacturers were obtained directly from the manufacturers. For each food type, two independent batches were analysed in triplicate (deviating from the EURL guidelines), except for goat’s milk cheese, where three independent batches were used. All the foods were within their shelf-life and tested for the presence of *L. monocytogenes* before use, and at the end of the experiment, using the standard method ISO 11290-1 [32].

### 4.4. Inoculation of the Food

Except for coleslaw, each food sample was cut into approximately 11 pieces of 25 g each. Cheese and paté were cut into cubes whereas smoked salmon was in folded slices. In total, 240 µL of the inoculum was divided by the number of sides to be inoculated and the appropriate volume was spread on each side using a sterile plastic loop. The total inoculum was < 1% of the weight of the sample. The samples with some sides inoculated were left to dry in the laminar air flow cabinet for one minute, before inoculating the other side(s). The objective was to get a total inoculum of 100 cfu g^−1^. One sample of each food was enumerated immediately using the standard method ISO 11290-2 [31] and the remainder were individually vacuum packed in shrink bags (Caterlite DM065, Bristol, UK) prior to storage. For the coleslaw, a fine spray of the suspension of inoculation was mixed with the coleslaw at 1% of the weight of the coleslaw to give a target inoculum of 100 cfu/g, following which the coleslaw was sub-divided into sterile containers containing 25 g each. The samples were incubated at different temperatures for different lengths of time, as follows: Smoked salmon was incubated at 6 °C for 5 days and 8 °C for 9 days; cheese and pork pate were incubated at 8 °C for 14 days (although the pork pate experiment was stopped after 7 days as growth was observed); and the coleslaw was incubated at 8 °C for 7 days and 12 °C for 14 days.

### 4.5. Analysis for L. monocytogenes

*L. monocytogenes* detection and enumeration was undertaken following the EN ISO 11290-1 and ISO 11290-2 methods, respectively, only using chromogenic agar [31,32], and pour-plating 10 mL of the initial dilution of the food on a 140 mm Petri dish to increase the sensitivity of the assay 10-fold, detecting > 1 cfu g^−1^ [33]. Uninoculated food samples were tested by enrichment only and inoculated food samples were enumerated. For all foods, a 25 g portion was diluted in 225 mL of MRD and mascerated in a stomacher for 3 min. On day 0, a 2.5 mL portion of this dilution was spread on a 140 mm chromogenic agar plate (Agar Listeria according to Ottaviani and Agosti; ALOA) and 0.5 mL was also spread on a 90 mm plate. On day 7 and day 14, the same dilutions were used and additional serial dilutions were also used to obtain a countable bacterial number. All agar plates were incubated at 37 °C for two days.

### 4.6. pH and Water Activity Measurements

pH and water activity were measured the first day and at day 14. For the pH, 20 g of sample was homogenised with 12 mL of distilled water in a stomacher for 3 min. The pH was measured with a pH probe (Hanna pH 211). The water activity was determined using an Aqualab model Series 3TEB water activity meter (following the manufacturer’s instructions).

### 4.7. Total Bacterial Count Determination

The total bacterial count (TBC) was obtained for each food at day 0 and day 14. Twenty-five grams of food was diluted with 225 mL of MRD, homogenised in a stomacher for 3 min and serially diluted in MRD to 10^−7^. A volume of 0.1 mL of an appropriate dilution was spread on a plate count agar (PCA) plate. The plates were incubated at 30 °C for 24 h and counted.

### 4.8. Calculation of Growth Potential (δ)

The *L. monocytogenes* numbers at each time point were determined and the log_10_ of the number was calculated. The *δ*, which represents the difference between the number of *L. monocytogenes* at the end and at the beginning or middle of the experiment, was calculated for each experiment: *δ = log_10_ cfu g^−1^ end or middle of the challenge study—log_10_ cfu g^−1^ beginning of the challenge study;*

If δ ≥ 0.5 log_10_ cfu g^−1^, then it is considered that the food supports *L. monocytogenes* growth.

### 4.9.Statistical Analysis

According to the EURL guidelines, for the determination of δ of *L. monocytogenes*, the worst-case scenario is used. Hence, all the data points are shown.

For TBC, pH, and water activity, the results from each food type were averaged. For comparison of the results between the beginning and end, an independent 2 sample equal variance, 2 tail *t*-test using excel was conducted. Significance was determined at *p* < 0.05.

## 5. Conclusions

Following the EURL guidelines on challenge studies to determine the growth of *L. monocytogenes* in food is beneficial. The results from future studies can be compared if standard methods are used. It is evident from this study that there are factors in food other than pH, water activity, and TBC that influence the ability of *L. monocytogenes* to grow in food.

## Figures and Tables

**Table 1:** The growth potential (δ) of *L. monocytogenes* in various foods. The potential to grow is based on a worst-case-scenario where the numbers at the middle or end of the experiment are subtracted from the numbers at the beginning and growth is considered possible if any of the replicate values are >0.5 log cfu g^−1^.

Food	Batch	Temperature Profile of Incubation	*L. monocytogenes* Numbers (log cfu g^−1^)			Maximum log Difference at Day Middle	Maximum log Difference at Day End	Growth Potential (δ)
			Day 0	Day Middle	Day End			
Coleslaw	1	8 °C for 7 days and 12 °C for 14 days.	2.62; 2.30; 2.45 ^1^	0.82; 0.67; 0.80	Below the limit of detection ^2^	−1.63	−2.30	No
	2		2.54; 2.50; 2.51	0.80; 1.30; 1.37	Below the limit of detection	−1.20	−2.50	No
Feta cheese	1	8 °C for 14 days	2.14; 2.26; 2.38; 2.08	2.00; 2.00; 2.30; 2.30	Below the limit of detection	0.05	−2.10	No
	2		2.60; 2.56; 2.64; 2.34	2.38; 2.00; 1.90; 2.15	Below the limit of detection	−0.20	−1.00	No
Goat’s milk cheese	1	8 °C for 14 days	1.78; 1.90	Below the limit of detection; 0.90	Below the limit of detection	−1.00	−1.78	No
	2		1.90; 1.90	Below the limit of detection; 1.60	Below the limit of detection	−0.30	−1.90	No
	3		1.78; 1.48	Below the limit of detection	Below the limit of detection	−1.48	−1.48	No
Pork pate	1	8 °C for 7 days	1.75; 1.81	4.25; 4.47	ND ^3^	2.66	ND	Yes
	2		1.90; 1.90	4.87; 4.85	ND	2.97	ND	Yes
Smoked salmon A	1	6 °C for 5 days and 8 °C for 9 days	1.83; 1.68	1.64; 2.88	4.92; 5.19	1.20	1.71	Yes
	2		1.60; 1.51	3.10; 3.22	4.59; 3.92	3.27	0.71	Yes
Smoked salmon B	1	6 °C for 5 days and 8 °C for 9 days	1.90; 1.78	3.30; 3.21	3.81; 3.64	1.44	1.90	Yes
	2		1.90; 2.00	3.39; 2.83	3.60; 3.58	1.49	1.70	Yes

^1^ All the replicates are shown and are in sequential order at Day 0, Day Middle, and Day End. ^2^ The limit of detection was 1 cfu g^−1^. Unless otherwise shown as a number, this applies to all replicates. ^3^ Not determined.

**Table 2:** Total bacterial counts, pH, and water activity values of the food types at the beginning and end of storage. An * indicates a significant difference (*p* < 0.05).

Food	Total Bacterial Count (log cfu g^−1^)		Water Activity (a_w_)		pH	
	Day 0	Day End	Day 0	Day End	Day 0	Day End
Coleslaw	7.62 ± 0.160	7.50 ± 0.105	0.998 ± 0.001	0.985 ± 0.001 *	5.49 ± 0.465	6.51 ± 0.010
Feta cheese	7.33 ± 0.293	ND ^1^	0.973 ± 0.002	0.945 ± 0.018	4.58 ± 0.155	4.37 ± 0.150
Goat’s milk cheese	7.12 ± 0.366	8.22 ± 0.486	0.994 ± 0.002	0.986 ± 0.004 *	4.32 ± 0.058	4.15 ± 0.054 *
Pork pate	5.03 ± 0.271	7.58 ± 1.66 *	0.969 ± 0.004	0.964 ± 0.004	6.20 ± 0.134	5.89 ± 0.320
Salmon 1	4.74 ± 0.654	7.56 ± 0.911 *	0.979 ± 0.005	0.965 ± 0.002 *	6.12 ± 0.065	6.11 ± 0.098
Salmon 2	5.30 ± 0.455	7.83 ± 0.285 *	0.972 ± 0.004	0.950 ± 0.005	6.26 ±0.080	5.88 ± 0.110

^1^ Not determined.
